# Managing Depressive Symptoms in the Workplace Using a Web-Based Self-Care Tool: A Pilot Randomized Controlled Trial

**DOI:** 10.2196/resprot.7203

**Published:** 2017-04-04

**Authors:** Abigail Hirsch, Jason Luellen, Jared M Holder, Gregory Steinberg, Teresa Dubiel, Anna Blazejowskyj, Krista Schladweiler

**Affiliations:** ^1^ myStrength, Inc Denver, CO United States; ^2^ Centerstone of America Nashville, TN United States

**Keywords:** depression, behavioral health, health promotion, workplace, randomized controlled trial

## Abstract

**Background:**

Depression in the workplace creates a significant burden on employees and employers in terms of lost productivity and related costs. myStrength provides a robust, holistic Web- and mobile-based solution empowering users to learn, practice, and implement a range of evidence-based psychological interventions.

**Objective:**

The main aim of this study was to demonstrate improvement in depressive symptoms among employees at risk of depression through myStrength use.

**Methods:**

A 26-week, parallel-arm, pilot, randomized controlled trial was designed to assess the effectiveness of myStrength compared to a series of informational “Depression Tip/Fact of the Week” emails as the active control arm. Study participants (n=146) were commercially insured employees of a mid-sized financial software solutions firm. The primary outcome was self-reported change in depression score as best fit by a linear random effects model accounting for individual baseline symptoms.

**Results:**

The final sample consisted of 78 participants in the experimental arm, myStrength, and 68 participants in the active control arm. myStrength users demonstrated significantly steeper and more rapid reduction in depressive symptoms over time compared to the active control (*P*<.001), suggesting that the intervention generated improvement in behavioral health symptoms, even in a nonclinical sample.

**Conclusions:**

This pilot study builds foundational support for the scalable deployment of myStrength as a complementary behavioral health offering to promote overall mental health and well-being in the workplace.

## Introduction

Identifying tools to manage both clinical and subclinical depression in an effective and broad-reaching manner could have widespread benefits. Depression is one of the most common and debilitating disorders in the United States [1]. In 2014, 6.7% of American adults aged 18 years or older (15.7 million people) had at least one major depressive episode [2]. Such episodes can result in significant impairments in home life, work function, and social relationships.

Depression in the workplace poses a significant burden to both employees and employers. A nationally representative, epidemiologic survey found that 6.4% of employees in the workplace had depression; of those, 13.8% were classified mild, 38.5% moderate, and 47.7% as severe [3,4]. Depression is also a major cost driver for employers [5,6]. Depression annually costs US businesses over $51 billion in absenteeism from work and lost productivity on top of the $26 billion in direct treatment costs [7].

US employers looking for ways to manage the cost burden of depression in the workplace are increasingly turning to cutting-edge platforms to provide real-time access to care [8,9]. One way to bend the cost curve is to broaden the reach of preventative interventions to engage people both with clinical levels of depression and those demonstrating subthreshold depressive symptoms before symptoms and associated costs elevate [10-13].

One cost-effective way to provide such preventative care for depression is via Web-based interventions [14,15]. Computerized cognitive behavioral therapy (cCBT) for treating clinical depression has been well validated in efficacy trials [12,16]. Likewise, randomized controlled trials (RCTs) suggest that the positive impacts from online tools for people diagnosed with clinical depression also extend to those with milder symptoms [17,18]. Interestingly, a growing body of literature suggests that the greatest impact of cCBT is found among users who might not seek out or have access to other standard treatment options like talk therapy [19-21].

In developing an appropriate digital platform to be used for broad, cost-effective, employer-based outreach, scalability and low price point per user are paramount. At the same time, past research suggests that having a guided component to Internet interventions improves engagement and outcomes [22,23]. In an era where Web and mobile apps can be customized to the individual user, it is possible that such customization could help to increase engagement and impact without elevating program costs as with guided interventions.

myStrength, the Web and mobile self-care platform piloted in this study, is designed to close the behavioral health care treatment gap by extending access to empirically validated psychological interventions through a population-based approach [24]. myStrength builds on existing Web-based cCBT for clinical depression by incorporating wrap-around brief resources from mindfulness approaches, acceptance and commitment therapy, brief strategic therapies, and motivational interviewing while offering a unique set of programmatic recommendations to each user based on a profile process. The population health approach taken allows for the deployment and evaluation of psychological interventions in a real-world setting for employer-based depression management.

For the past 6 years, myStrength has been delivering evidence-based psychological interventions to users affiliated with over 100 mental health providers, ranging from large national health insurance providers to community mental health clinics. This pilot study is the first of its kind to rigorously evaluate the impact and effectiveness of myStrength in terms of depressive symptom burden reduction in a real-world setting.

## Methods

### Study Design

**Figure 1:**
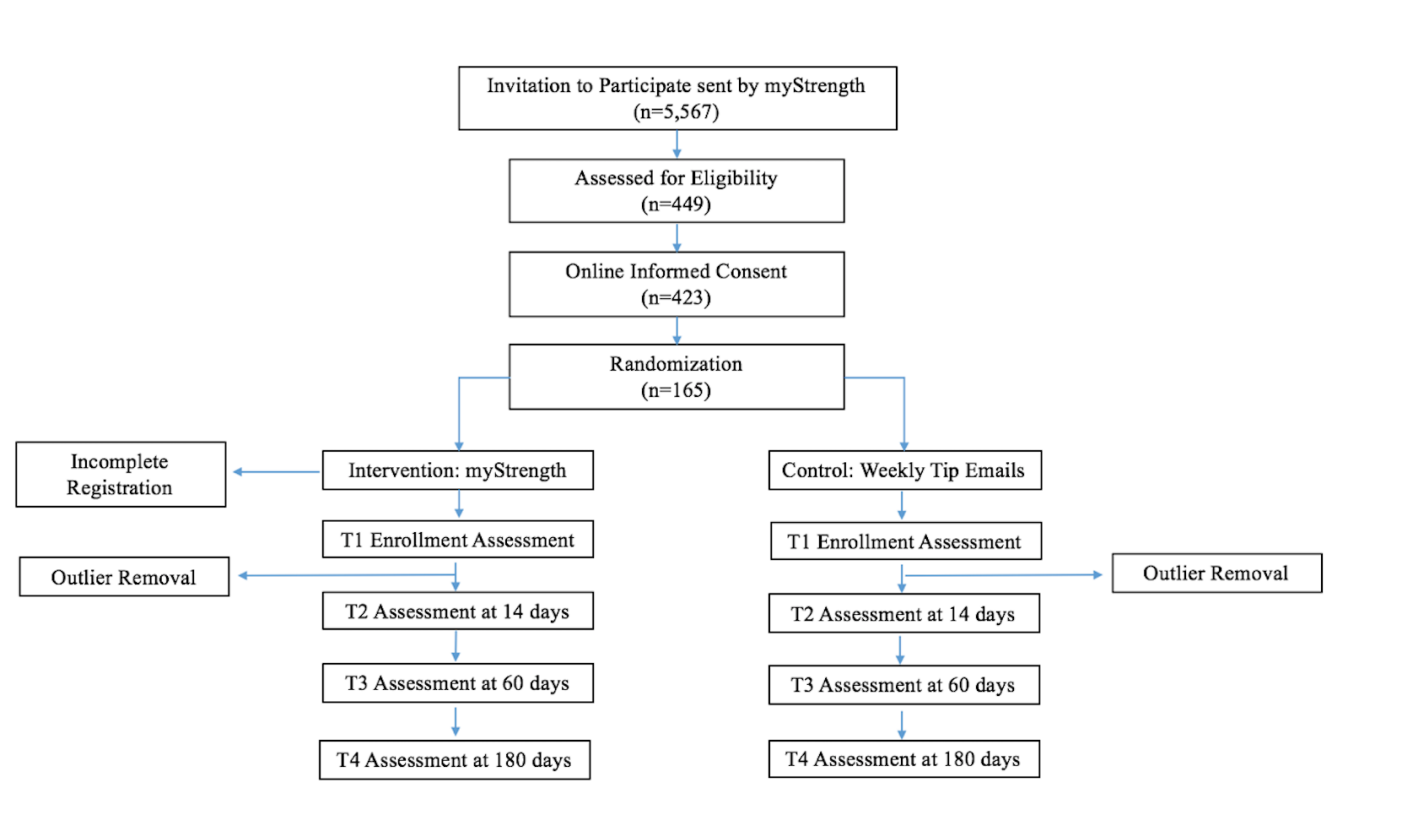
Study flowchart.

A 26-week, parallel-arm RCT with a single pretest and three posttests was piloted to examine the impact of myStrength use on self-reported depression symptom burden. This design was selected in accordance with the Pretest-Posttest Control Group Design with Multiple Substantive Posttests recommended by Shadish et al [25]. See the study flowchart in Figure 1, including an overview of the enrollment process and assessment timing.

### Study Participants and Recruitment

Participants were recruited from the 5567 commercially insured employees of a financial software solutions company. The study was conducted in partnership with Aetna, the company’s health benefits provider, and was open to all employees. Participants were excluded if they were under 18 years of age; were diagnosed with Alzheimer's disease, Parkinson's disease, dementia, delirium, or other cognitive impairment; had been institutionalized; or were diagnosed with obsessive-compulsive disorder because in each of these cases there were concerns raised by the sponsoring entity about the potential for adverse events in an unmonitored setting.

An executive recruitment email was sent out to all employees by the company’s head of human resources inviting individuals to voluntarily participate in the pilot of new tools for managing mood and stress. Two follow-up recruitment emails were sent out, with 5 days between each email. Participants were told there were two arms of the study intended to improve mental health during informed consent: (1) an interactive website and (2) receipt of an email series.

Interested participants accessed an online enrollment screener and completed informed consent. On the first page of this screening, potential participants were asked if they met any of the exclusion criteria. Those who endorsed exclusion criteria were redirected to a general resources page and not permitted to continue registration for the study. Because each employee received a unique email link to the registration survey, this redirect also meant people could not return and alter their responses.

### Intervention Group

The experimental arm was given access to myStrength resources include the following: cCBT modules for depression and anxiety, mindfulness and other empirically validated tools taught via short form video, substance use motivational interviewing and relapse prevention modules, a mood tracker, community and personal inspirations, motivational quotes, spiritual and faith-based resources if users elect to receive these, and a searchable library of over 1600 additional mental health and wellness resources if users elect to receive these, and a searchable library of over 1600 additional mental health and wellness resources. In addition, participants in the experimental arm received a series of 12 emails highlighting different aspects of myStrength and specific tools for managing depression, as well as reminders to access the platform. Participants could access any of the resources available on the myStrength website and mobile platform at their own pace and at any time during the 26-week study period. Multimedia Appendices 1 and 2 provide visual representations of the myStrength Web and mobile interface.

### Active Control Group

The active control arm received 12 “Tip/Fact of the Week” emails with factual statements about mental health or depression during the 26-week study period. Emails were designed to contain accurate information pertaining to depression signs and symptoms without offering clinical suggestions or advice (for example, see Multimedia Appendix 3).

### Outcome Measures

The primary outcome measure was the depression subscale score (range 0 to 42) of the Depression, Anxiety, and Stress Scale (DASS-21), a 21-item validated self-report questionnaire designed to measure symptom severity [26]. Participants in both the experimental and active control arms followed the same assessment schedule. Each participant completed an assessment at enrollment (T1) and were prompted by email reminders to be reassessed at 14, 60, and 180 days (T2-T4, respectively). Demographic information collected included gender, age, level of education, ethnicity, and mental health treatment status.

### Randomization and Blinding

A randomized design was selected to prospectively assess the impact of myStrength use on the primary outcome, depression score over time. The online survey software, FluidSurveys, consented and randomly assigned study participants to either the intervention arm or active control. Per institutional review board (IRB) review and approval, study participants knew of their study arm assignment; however, they were unaware that the active control did not include platform access. Randomization occurred after prescreening for eligibility and informed consent and thus recruited a study population more open to help-seeking. The study employed a sampling ratio of 1.5:1 with no stratification and with the larger number of individuals assigned to the experimental arm. This approach was taken to sufficiently power the experimental arm, as those study participants engaged with myStrength were required to complete the extra step of accessing the website and completing registration for the program. The active control arm had no similar post-enrollment burden.

### Statistical Analyses

Data analyses were conducted using R version 3.3.2 (The R Foundation). The primary outcome, depression score, was analyzed using mixed effects regression. A random-intercept was modeled to account for individual baseline variation. Predictors included study arm (group), a continuous measure of time (days since baseline; time), and the interaction of group and time (group × time). Covariates included gender, age group, and a baseline indicator of whether the participant was receiving treatment outside the study as measured by self-report at T1 (receiving treatment). In addition, each regression model adjusted for the corresponding baseline T1 outcome assessment (depression).

### Ethics

All data collected were retained by the experimenters and no individual-level data were shared with Aetna Inc or the employer. Likewise, myStrength, the intervention offered in the experimental arm, stores all responses in a manner compliant with the Health Insurance Portability and Accountability Act.

This pilot study was not prospectively registered on Clinicaltrials.gov given the original intent to demonstrate feasibility. This research was reviewed and approved by Sterling IRB #4959.

## Results

### Participant Flow and Outlier Consideration

As depicted in Figure 1, the recruitment email was sent out to the census of 5567 employees. Among them, 449 (8.1%) completed the online enrollment screening, 26 (5.8%) of whom did not meet eligibility criteria and were excluded. The randomization sample comprised 165 screened individuals (39.0%) who met the eligibility criteria and signed informed consent to participate. Randomization resulted in 96 participants in the experimental arm (58.2%) and 69 participants in the active control arm (41.8%).

Of 96 experimental study participants, 12 (12.5%) failed to complete registration. Case review revealed their data to be particularly sparse with regard to outcomes and covariates. Only 3 of 12 had any outcome data available, typically a single response on the third occasion. For that reason, study participants who failed to complete registration were omitted from the primary analyses, and the effective sample size was reduced from 165 to 153. Acknowledging the *a priori* goal of analyzing the data by intention to treat, this pilot study was unable to impute the missing data due to sparseness.

Despite random assignment to study group, between-group differences on the DASS-21 depression score at baseline (T1) were identified and subsequently removed from all analyses. A total of 7 statistical outliers (6 in the experimental group and 1 in the active control group) were identified as 1.5 *I* beyond the upper or lower quartile, where *I* is the interquartile range. Descriptive statistics and regression models were conducted with and without the inclusion of outliers and the interpretation of findings was consistent.

**Figure 2:**
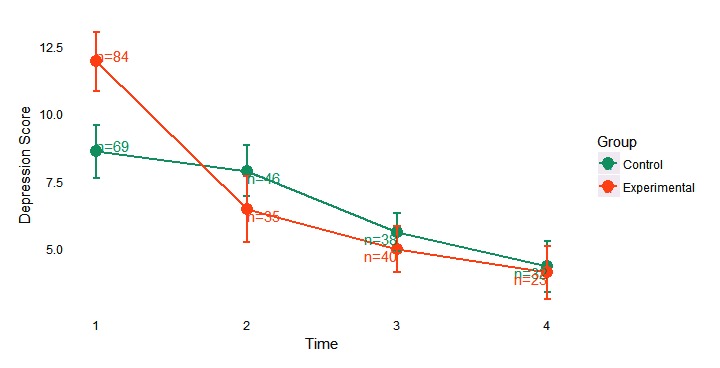
Depression scores over time, stratified by group, with outliers included.

**Figure 3:**
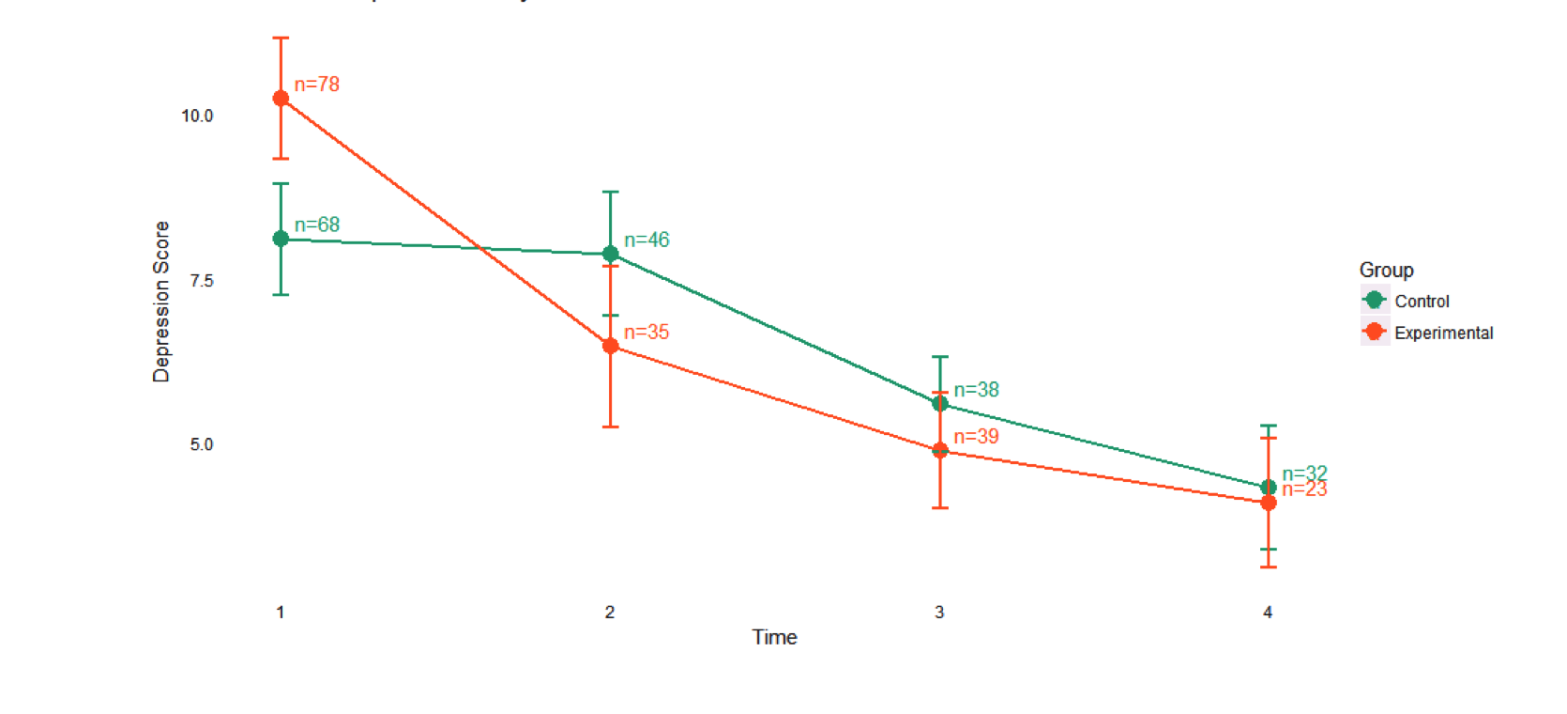
Depression scores over time, stratified by group, with outliers removed.

To demonstrate the impact of outlier inclusion on the average depression score over time, a side-by-side comparison of the study population with and without the outliers identified at baseline is presented. Figure 2 shows the average depression score over time for both the experimental and active control arms with the 7 statistical outliers included. Figure 3 shows the average depression score over time for both the experimental and active control arms with the outliers removed. Clear separation in depression scores was observed between the experimental arm and active control at baseline; however, the experimental arm with the outliers included (n=84) is skewed in favor of more severe depressive symptom burden as compared to the trimmed experimental arm with the outliers removed (n=78). Given the intent of the pilot study to assess the feasibility of myStrength to favorably impact depressive symptoms in a relatively healthy employee population, the statistical outliers were removed from all further analyses.

**Figure 4:**
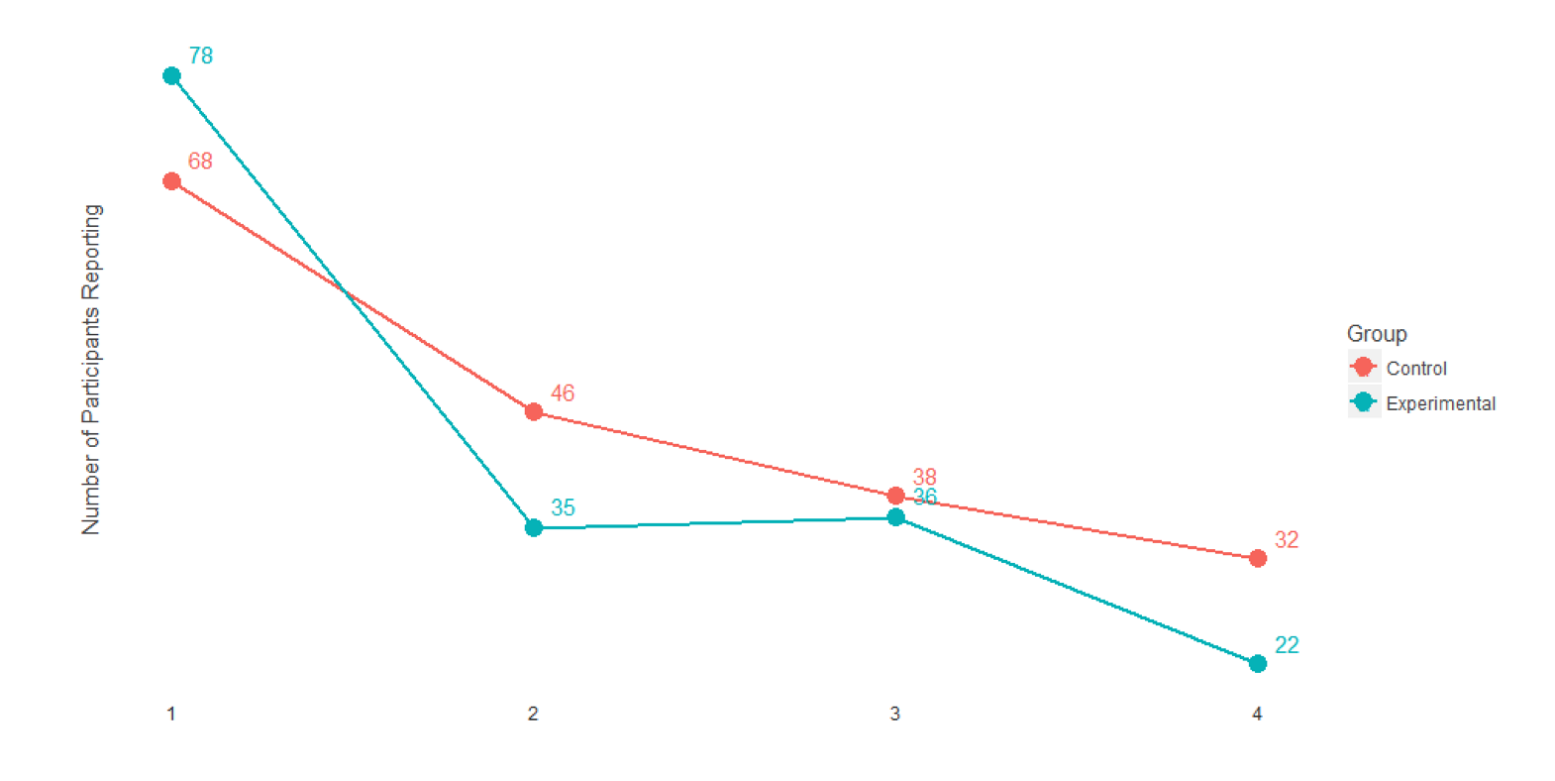
Number of participants who completed assessments during the study period, stratified by group.

Efforts were made to maintain participant engagement throughout the study and collect data for all measures on all occasions. Recent research on Internet-based depression interventions report variable adherence to study arm protocols and often experience high dropout rates [27,28]. Melville et al [27] synthesized 19 peer-reviewed studies referencing attrition among Internet-based behavioral health treatment interventions. Study dropout rates ranged from 2% to 83% and on average, 31% of study participants prematurely exited studies. Given that the pilot study described here was intended to mirror an unguided, real-world design, there was no human outreach component or offer of any financial incentive to increase study participation. Data were missing due to intermittent responses as well as some permanent dropout; all partial data were included in analyses. The overall attrition rate was 62% with 72% of experimental and 53% of the active control observations missing. Figure 4 shows the number of completed assessments in the experimental and active control groups.

### Demographic Characteristics

Among the experimental study arm, 75.3% were between 41 and 60 years old, 53.3% were female, 93.5% were Caucasian, and 64.9% were not receiving any mental health treatment. Among the active control arm, 60.3% were between 41 and 60 years old, 57.4% were female, 85.3% were Caucasian, and 60.3% were not receiving any mental health treatment. See Table 1 for the complete demographic characteristics, stratified by group, and for the overall study population.

**Table 1:** Demographic characteristics of participants (n=145; note: one participant did not answer any demographics questions and was excluded from this table).

Characteristics		Experimental n (%)	Control n (%)	Overall n (%)
**Age group**				
	18-40 years	15 (19.5)	20 (29.4)	35 (24.1)
	41-60 years	58 (75.3)	41 (60.3)	99 (68.3)
	61+ years	4 (5.2)	7 (10.3)	11 (7.6)
**Gender**				
	Female	41 (53.3)	39 (57.4)	80 (55.2)
	Male	36 (46.8)	29 (42.7)	65 (44.8)
**Ethnicity**				
	Caucasian	72 (93.5)	58 (85.3)	130 (89.7)
	African American/black	2 (2.6)	2 (2.9)	4 (2.8)
	Hispanic	1 (1.3)	2 (2.9)	3 (2.1)
	Other	2 (2.6)	6 (8.8)	8 (5.5)
**Receiving treatment**				
	No	50 (64.9)	41 (60.3)	91 (62.8)
	Yes	27 (35.1)	27 (39.7)	54 (37.2)

### Primary Analyses

The primary outcome of interest is change in depression score over time. As shown in Table 2, both the experimental and active control study participants experienced a reduction in depressive symptoms over time. Given that group membership was assigned after informed consent was obtained, it is hypothesized that the pilot study population was more open to help-seeking/treatment than the general population. Also, depressive symptoms, especially mild ones, are well established to eventually decrease even without intervention on their own over time. On average, study participants in the intervention group accessed the myStrength platform 6.09 times during the 26-week study period.

**Table 2:** Descriptive statistics for depression by group and time.

Outcome		n	Mean	SD	95% CI
**Experimental**					
	Time 1	78	10.2	8.1	8.40-12.1
	Time 2	35	6.5	7.3	3.96-9.0
	Time 3	36	4.8	4.8	2.84-6.7
	Time 4	22	4.0	4.8	1.86-6.1
**Control**					
	Time 1	68	8.1	7.0	6.40-9.8
	Time 2	46	7.9	6.4	5.98-9.8
	Time 3	38	5.6	4.4	4.14-7.0
	Time 4	32	4.3	5.3	2.40-6.2

The rate of depression symptom burden improvement was further tested by linear mixed effects regression modeling. A random intercept model was selected to account for individual baseline depression symptoms, and model findings are summarized in Table 3. The intercept is statistically significant, underscoring the importance of taking into account personal depression trajectories (*b* coefficient=4.27, *P*<.001). Receiving mental health treatment, such as outpatient therapy or taking antidepressant medication, was found to be an independent predictor of depression score reduction over time (*b* coefficient=–1.13, *P*=.026). The main effect of time was found to be statistically significant, meaning that in general, a 0.83 reduction in depression score was achieved at each time point (*b* coefficient=–0.83, *P*=.002). This main effect finding is qualified by the interaction group × time (*b* coefficient=–1.35, *P*<.001). Taking into account potential confounders, the experimental arm experienced an accelerated trajectory of depression symptom reduction to a factor of 1.35 times faster than the active control arm.

**Table 3:** Summary of mixed effects regression analysis predicting depression.

Effect	Estimate	SE	Degree of freedom	*t* value	*P* value^a^
Intercept	4.27	0.81	303.00	5.26	<.001
Group (experimental)	1.53	0.93	333.60	1.64	.101
Time	–0.83	0.27	296.60	–3.08	.002
Gender (female)	–0.63	0.47	154.20	–1.33	.187
Age group (41-60 years)	–0.29	0.55	149.80	–0.53	.595
Age group (61+ years)	–0.15	0.84	119.20	–0.17	.863
Receiving treatment	–1.13	0.50	158.70	–2.24	.026
Depression baseline	0.72	0.03	146.70	23.56	<.001
Group × time (interaction)	–1.35	0.39	308.90	–3.42	<.001

^a^Satterthwaite approximation was used to estimate degrees of freedom and estimate *P* values. Regression estimates are unstandardized.

## Discussion

### Principal Findings

Findings from this pilot study support the feasibility of delivering impactful Web- and mobile-based depression interventions directly to employees. This innovative partnership brought together a large commercial health insurance provider, a mid-sized US corporation, and a self-help platform provider of behavioral health and wellness to empower employees with effective self-care tools.

Principal findings suggest that while participants in both study arms experienced improvement in depression symptoms, the experimental arm achieved those favorable results faster and to a greater degree than the active control arm. Within 2 weeks of study enrollment, depression score differences between the two groups emerged with more rapid improvement over time for those participants in the experimental arm. While mean symptom levels began to converge by the 6-month assessment, study participants in the experimental group spent less time being symptomatic compared to the active control group.

In addition, this research supports the premise that evidence-based Web and mobile behavioral health interventions are a viable way to reach employees coping with depressive symptoms who might not otherwise seek out behavioral health care. As seen in Table 1, almost two-thirds of all study participants received no mental health treatment, despite being identified as at risk of depression. This pilot study demonstrated the ability to reach and impact a volunteer, help-seeking population who in the majority of cases were not using other available services. At the same time, the study showed considerable attrition over time. While we hypothesized that individual-level profile-based personalization of the program would drive retention, it appears further work is necessary to overcome this hurdle in order to maximize value from a low-cost, highly scalable, nonguided intervention.

### Limitations

This work has several notable limitations. First, due to the real-world, nonincentivized methodology, there was considerable attrition across the pilot study. Barring imputation, we employed statistical approaches that model and control for such attrition; however, it is always possible that subjects lost to follow-up were qualitatively different from those providing follow-up data. If nonresponders in the experimental arm were more likely to drop out of the study, this would certainly have biased the results. Future research building on this pilot study will seek to balance the real-world intent of the program with the possibility of offering incentivized outbound calling or weekly text message reminders to improve study retention.

Selection of a proper control condition is always challenging in behavioral health research. Because participants in such behavioral health research inherently know their assigned intervention arm (myStrength or email series in this case), a well-designed study must incorporate an active control that is equally appealing to participants as the intervention, while not containing the key active ingredients of the intervention [29]. One measure of the effectiveness of an active control is to look for between-group differences in terms of satisfaction with the treatment provided. We asked all participants to rate their satisfaction in each study arm at each reassessment interval. Overall, 93% of study participants in the experimental arm and 73% of study participants who received the active control reported that they were engaged or highly engagement in email campaigns. Given the high satisfaction rates across both groups, the active control email series was considered to be a viable control condition. It was hypothesized that depressive symptom improvement would be achieved in both the experimental and active control groups, given the episodic nature of depression. Research findings substantiated this hypothesized outcome.

In addition, it is possible that myStrength may have variable impact on depression scores among real-world users. Study participants in the experimental arm were required to complete informed consent to participate in this pilot program and theoretically may have been more motivated to use and benefit from such a platform. With only one-third of the sample employee population opting to complete informed consent, we have no additional information on those who chose not to participate to test for selection bias. Building on these early findings, an observational study is planned to understand the real-world uptake and use of myStrength to minimize this bias.

Finally, the identification and removal of statistical outliers in both study arms at baseline (T1) warrants acknowledgment. Following widely accepted measurement methodology for the identification of outliers, the removal of 7 total outliers was justified. However, the fact that the experimental arm overindexed in outliers might suggest that randomization failed to generate completely equivalent groups—an occurrence that can happen despite randomization. Given the small, pilot nature of this study, future research will employ larger sample sizes to avoid this potential shortcoming.

### Conclusions

The results of this pilot study highlight the promise of incorporating self-care tools such as myStrength into the portfolio of behavioral health care offerings. Web and mobile tools are considered complementary, add-on offerings and are not intended to replace face-to-face therapy but rather to expand access to care and to deliver services with lower barriers to entry directly to employees. These preliminary study findings support larger scale efforts to evaluate if employers and their employees could benefit from incorporating an easily accessible and readily available self-care platform to address behavioral health needs.

The myStrength platform may serve as a “just in time” responder preventing mild symptoms from erupting into full-blown clinical depression while easing entry into professional care for those employees grappling with the perceived stigma around diagnosis and seeking treatment. Likewise, the results presented here also suggest that Web and mobile platforms may be a powerful tool for reducing productivity loss due to subclinical behavioral health concerns. myStrength provides low-threshold access to information and evidence-based tools, which could help employees immediately in the short term while also, where appropriate, guiding employees to recognize the need for additional care and avoid costly treatment in the long term.

## Appendix

Multimedia Appendix 1 myStrength Web-based log-in interface.

Multimedia Appendix 2 myStrength mobile interface.

Multimedia Appendix 3 Active control informational email example.

## Abbreviations

cCBT: computerized cognitive behavioral therapy
DASS-21: Depression, Anxiety, and Stress Scale with 21 items
IRB: institutional review board
RCT: randomized controlled trial
