# Supplementary figures and images for: Managing Depressive Symptoms in the Workplace Using a Web-Based Self-Care Tool: A Pilot Randomized Controlled Trial
Source: JMIR Res Protoc. 2017 Apr 4;6(4):e51. doi: 10.2196/resprot.7203 (PMC5395692; doi:10.2196/resprot.7203)

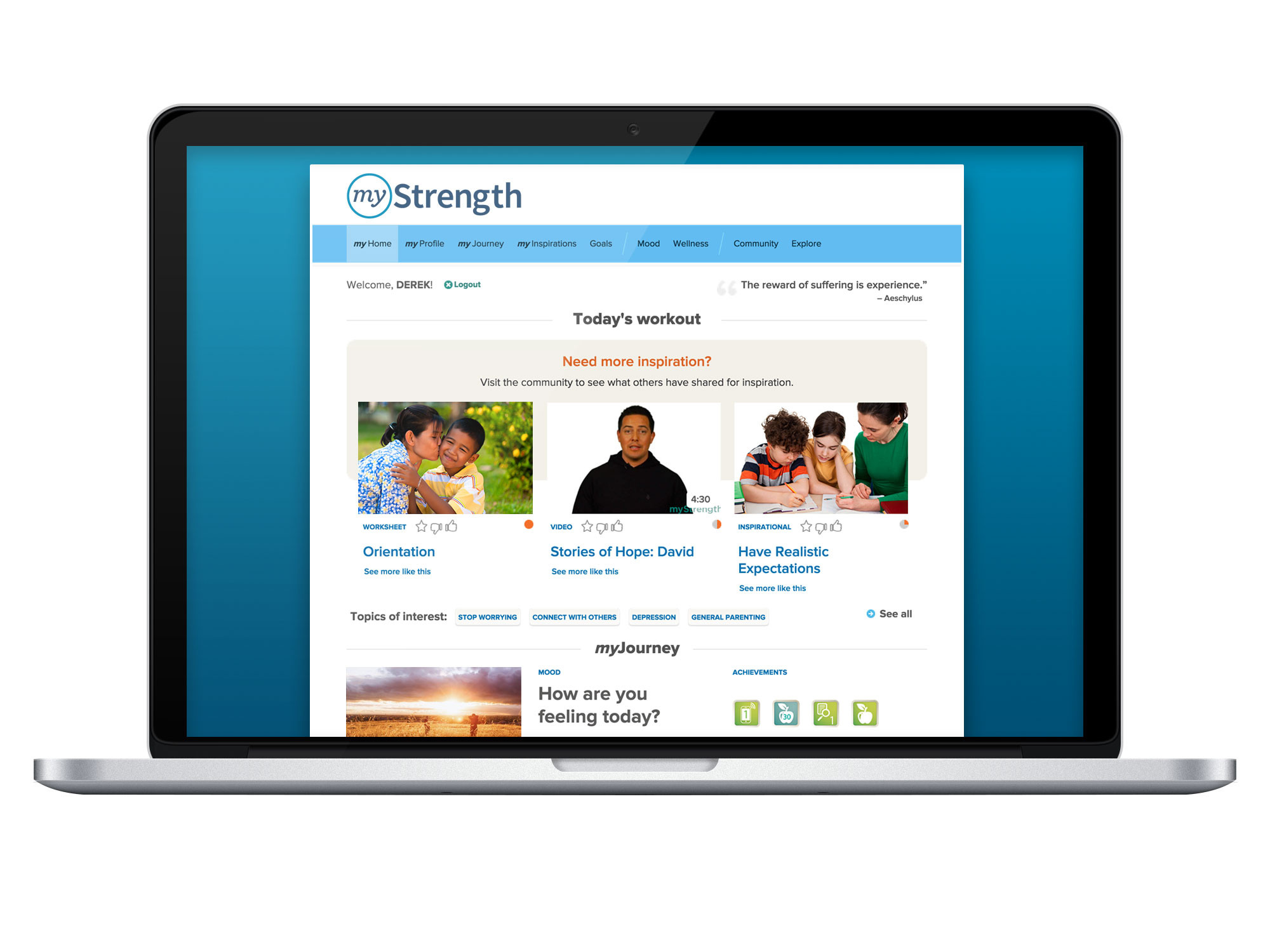

Supplement: Multimedia Appendix 1 [file resprot_v6i4e51_app1.jpg]

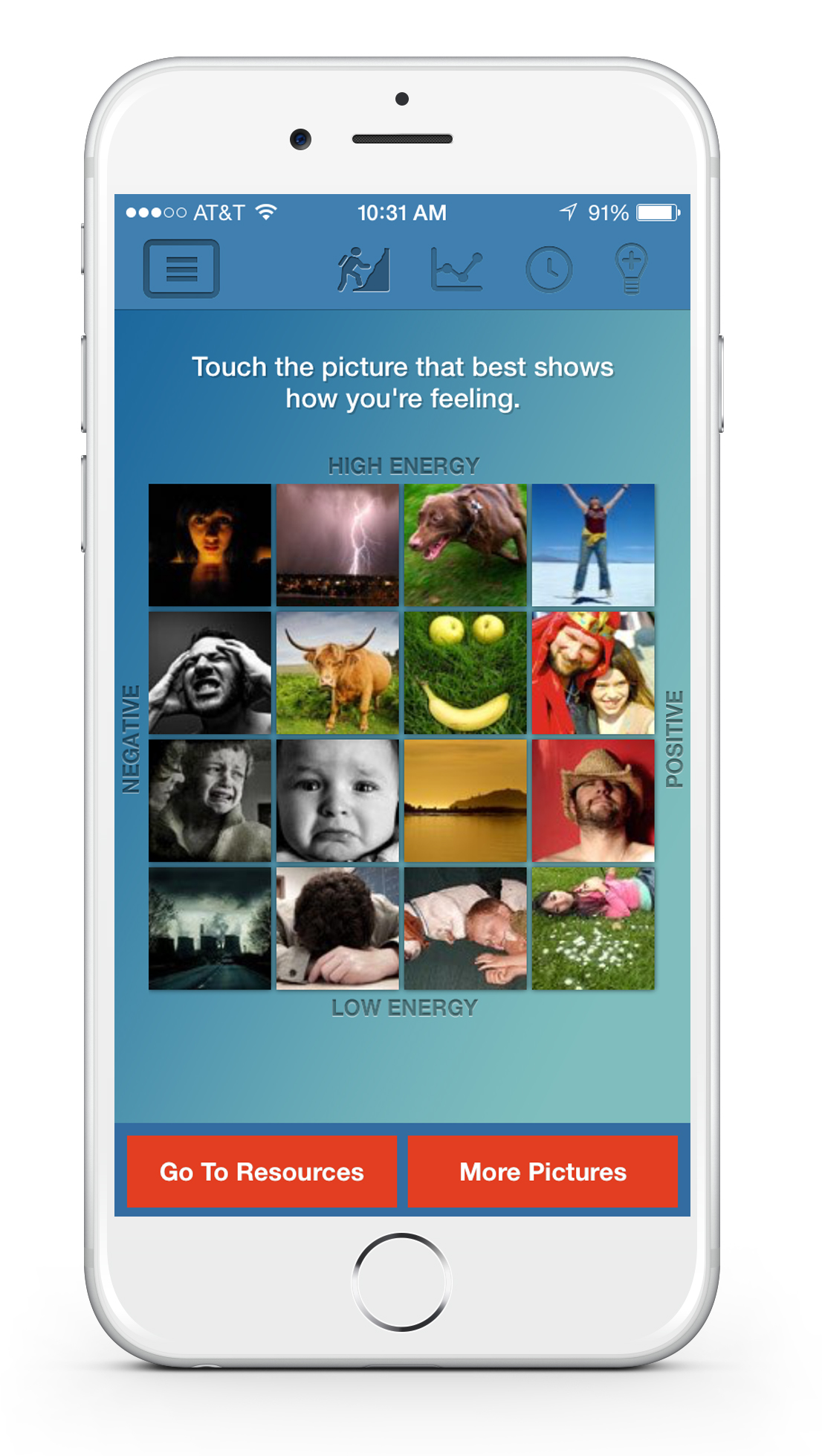

Supplement: Multimedia Appendix 2 [file resprot_v6i4e51_app2.jpg]

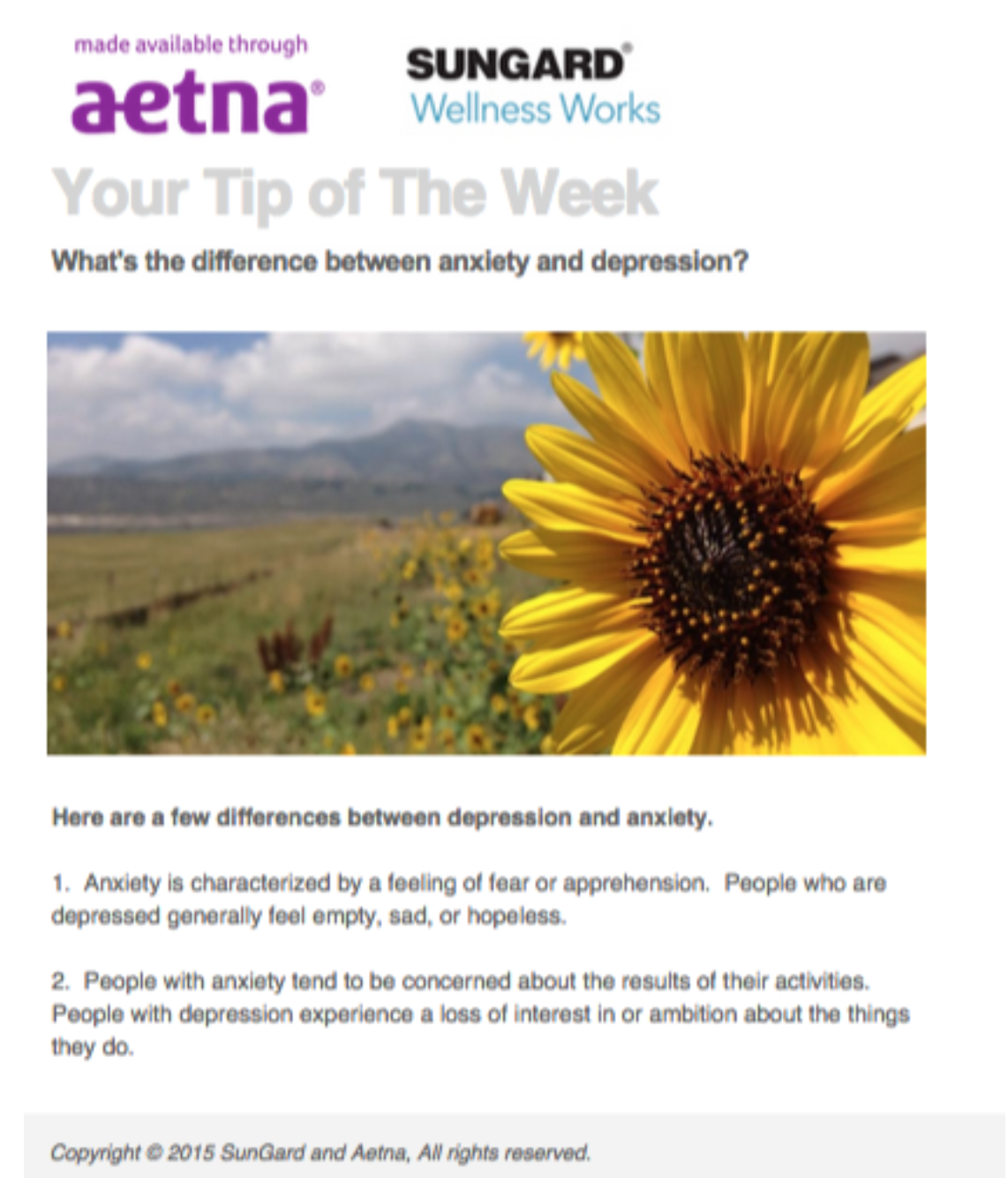

Supplement: Multimedia Appendix 3 [file resprot_v6i4e51_app3.png]
